# Antibiofilm activity of ultra-small gold nanoclusters against *Fusobacterium nucleatum* in dental plaque biofilms

**DOI:** 10.1186/s12951-022-01672-7

**Published:** 2022-11-03

**Authors:** Yangheng Zhang, Rixin Chen, Yuxian Wang, Peng Wang, Jiajie Pu, Xiaoqiang Xu, Faming Chen, Ling Jiang, Qing Jiang, Fuhua Yan

**Affiliations:** 1 Nanjing Stomatological Hospital, Medical School of Nanjing University, Nanjing, 210008 China; 2 College of Food Science and Light Industry, State Key Laboratory of Materials-Oriented Chemical Engineering, Nanjing Tech University, 211816 Nanjing, China; 3 State Key Laboratory of Pharmaceutical Biotechnology, Department of Sports Medicine and Adult Reconstructive Surgery, Nanjing Drum Tower Hospital, The Affiliated Hospital of Nanjing University Medical School, Nanjing, 210008 China; 4 01life Institute, 518000 Shenzhen, China; 5 State Key Laboratory of Military Stomatology & National Clinical Research Center for Oral Diseases & Shaanxi Clinical Research Center for Oral Diseases, Department of Periodontology, School of Stomatology, Fourth Military Medical University, Xi’an, 710032 China

**Keywords:** Gold nanoclusters, Antibiofilm activity, Dental plaque, *Fusobacterium nucleatum*, Microbiome

## Abstract

Pathogenic dental plaque biofilms are universal and harmful, which can result in oral infections and systemic diseases. Many conventional therapeutic methods have proven insufficient or ineffective against plaque biofilms. Therefore, new strategies are urgently needed. *Fusobacterium nucleatum* (*F. nucleatum*), a periodontal pathogen associated with a variety of oral and systemic diseases, is thought to be central to the development and structure of dental plaques. Here, ultra-small gold nanoclusters (AuNCs) were prepared. They exhibited potent antibacterial activity against *F. nucleatum* through enhanced destruction of bacterial membranes and generation of reactive oxygen species. Furthermore, due to their excellent penetration, the AuNCs could inhibit biofilm formation and destroy mature biofilms in vitro. Their antibiofilm efficacy was further confirmed in a mouse model, where they reduced biofilm accumulation and ameliorated inflammation. Meanwhile, the disruption of oral and gut microbiota caused by colonization of oral *F. nucleatum* could be partially restored through AuNCs treatment. Therefore, AuNCs could be considered as promising antibiofilm agents and have great potential in the clinical treatment of dental plaque.

**Graphical Abstract:**

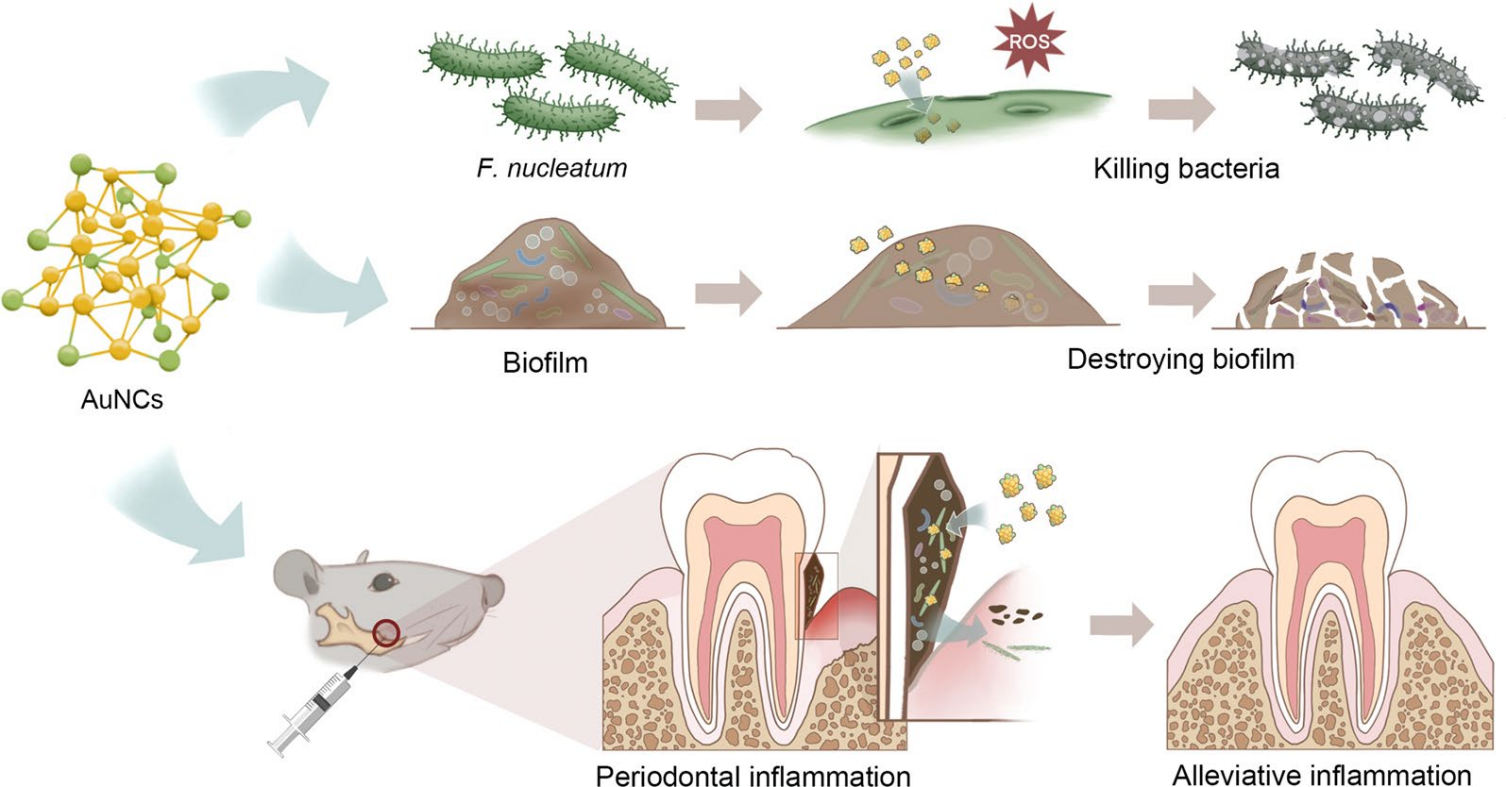

**Supplementary Information:**

The online version contains supplementary material available at 10.1186/s12951-022-01672-7.

## Introduction

The bacterial populations of the mouth are extremely complex with approximately 700 species [1]. They live primarily as polymicrobial biofilms on mucosal and dental surfaces [2]. Oral biofilms are physiologically and structurally organized groups of microbial communities that are embedded in an extracellular matrix [2]. The formation of pathogenic biofilms, such as *Streptococcus mutans*, *Porphyromonas gingivalis* and *Aggregatibacter actinomycetemcomitans*, could cause dental caries, periodontitis and peri-implantitis [2]. They may also influence the initiation and progression of numerous systemic diseases [3]. Therefore, controlling pathogenic dental plaque is of clinical significance for both oral and systemic health.

Studies on the oral biofilm have shown that *Fusobacterium nucleatum* (*F. nucleatum*) serve as the key orchestrator of biofilm formation and maturation [4]. During the formation and subsequent growth of biofilm, *F. nucleatum* connects primary colonizers such as *Streptococcus* species to the latter pathogenic bacteria [5]. By this way, *F. nucleatum* could facilitate the survival and growth of some key pathogen, such as *P. gingivalis* and *A. actinomycetemcomitans* [4, 5]. *F. nucleatum* could further shape host responses and enhance other oral pathogens’ pathogenicity [6, 7]. In addition, *F. nucleatum* has aroused attention in recent years because of an increasing number of associations with extraoral diseases [5, 8]. Targeted elimination of *F. nucleatum* by vaccines or phages could reduce pathogenic bacterial co-aggregation and biofilm formation have been confirmed in some studies [9, 10]. Thus, treatment and eradication of *F. nucleatum* could inhibit the development and reduce the pathogenicity of dental plaque biofilms.

The conventional treatment for dental plaque biofilms focuses on mechanical removal (supragingival and subgingival scaling) and antibiotics [11, 12]. However, mechanical removal may leave biofilm residues in some difficult-to-reach areas [11]. And the overuse and misuse of antibiotics will result in the emergence of multi-drug-resistant bacteria and the disruption of normal flora [13]. The development of nanotechnology has provided the potential for new treatment for biofilms. Nanoparticle coatings with antiadhesive property have been applied to the surfaces of medical instruments or implants [14, 15]. For the complex microenvironment of the oral cavity, nanoparticle drug delivery systems could be designed to enhance drug aqueous solubility and transport into bacterial cells [16, 17].Specially, the directly bactericidal activity of nanoparticles has broad application prospect in biofilm control [18, 19]. Among various types of nanomaterials, metal nanoparticles have attracted much attention due to native antibacterial properties and no drug resistance [18, 20]. Studies have confirmed that the activity of nanoparticles against biofilms is closely related to their properties, such as size, shape and crystalline structure [21].

Metal nanoclusters (MNCs) with ultra-small size (~ 2 nm) and precise simple structural composition are emerging as materials with antibacterial activity [22, 23]. Discrete electronic states and characteristic geometric structures confer several intriguing molecule-like properties to MNCs [23, 24]. Of the various MNCs, gold nanoclusters (AuNCs) have sparked increasing interest because of their biocompatibility and excellent antibacterial activity [25, 26]. AuNCs are eliminated completely through renal excretion within a few weeks of injection with no evidence of toxicity [25]. Additionally, the ultra-small size of AuNCs allows them to easily enter bacteria and disturb their metabolism [26], which was confirmed in our previous study [27]. It has also been reported that AuNCs affect intracellular redox signaling due to their intrinsic peroxidase-like catalytic activities [28, 29]. However, most studies have focused on the ability of AuNCs to kill common bacteria in planktonic states. It is still unclear whether ultra-small AuNCs are also effective against dental plaque biofilms.

In our previous work, AuNCs composed of 25 Au atoms and 18 thiolate ligands with ultra-small structure were prepared by a simple one-pot method [27]. We mainly focused on the evaluation of antibacterial efficiency of AuNCs toward Gram-negative and Gram-positive bacteria in vitro [27]. However, the antibacterial test of AuNCs on oral pathogenic bacteria and biofilm has not been reported. In this work, given the key role of *F. nucleatum* in dental plaque biofilms, the antibacterial effects of AuNCs on *F. nucleatum*-related biofilms were investigated in vitro and in vivo. The results indicated that AuNCs were effective against *F. nucleatum* in biofilms with good biocompatibility. Therefore, we hope this work can provide a guidance for the application of AuNCs in the treatment of dental plaque.

## Materials and methods

### Synthesis and characterization of AuNCs

6-mercaptohexanoic acid (MHA)-protected AuNCs were synthesized by one-pot reduction of sodium borohydride (NaBH_4_), as described in previous studies [27, 30]. The structure of AuNCs were characterized using a Lambda 25 UV/Vis Spectrophotometer (PerkinElmer, Waltham, MA, USA) and Nicolet iS500 Fourier Transform Infrared (FTIR) Spectrometer (Thermo Fisher Scientific, Waltham, MA, USA). The valence state and elemental composition of AuNCs were determined by X-ray photoelectron spectroscopy (XPS) using an ESCALAB 250Xi (Thermo Fisher Scientific). Their ultra-small sizes were observed by transmission electron microscopy (TEM) and high-resolution-TEM (HR-TEM) using HT7800 (Hitachi, Tokyo, Japan) and Tecnai G2F20 S-Twin (FEI, USA) microscopes, respectively. The zeta potential was measured by Zetasizer Nano ZS90 (Malvern Instruments, United Kingdom).

### Antibacterial effect of AuNCs in vitro

#### Plate counting assay

The culture method of *F. nucleatum* was describe in the Additional file 1.

The *F. nucleatum* suspension was treated with different concentrations of AuNCs (0, 0.1, 0.2, and 0.4 mM). After incubation at 37 °C for 6 h, the diluted bacterial suspensions were spread on the blood agar plates and cultured for 48 h. Percentage survival was evaluated according to the number of colonies on the plates with untreated bacteria as controls.

#### Bacterial membrane integrity

Membrane integrity was assessed by the LIVE/DEAD BacLight Bacterial Viability Kit (Thermo Fisher Scientific). *F. nucleatum* were treated with AuNCs (0.2 mM) or not for 6 h and then observed by Ti Eclipse Confocal Fluorescent Microscope (CFLM; Nikon, Tokyo, Japan) at magnifications of 60× following SYTO 9/propidium iodide (PI) fluorescent staining.

#### Scanning electron microscopy (SEM) and TEM

Briefly, bacteria treated with AuNCs (0.2 mM) or not were fixed with 2.5% formaldehyde and dehydrated with a graded ethanol series (30%, 50%, 70%, 80%, 90%, and 100%) for 15 min each. *F. nucleatum* were viewed under SEM (S-4700; Hitachi) or embedded in epoxy resin and cut into ultrathin sections for observation by TEM (HT7800).

#### Membrane potential test

Membrane potentials were determined using the fluorescent probe 3,3’-diethyloxacarbocyanine iodide (DiOC_2_(3)). *F. nucleatum* were treated as in the above experiment and exposed to 30 µM DiOC_2_(3) for 30 min at room temperature. Flow cytometry (BD FACSCalibur; Becton Dickinson, Franklin Lakes, NJ, USA) was used to analyze the samples using the 488-nm excitation laser and green and red filters to collect the data.

#### ROS detection

*F. nucleatum* were treated as described in the above experiment. Treated bacteria were incubated with a dichlorodihydrofluorescence diacetate (DCFH-DA) probe according to the manufacturer’s introduction and then observed by CFLM.

### Antibiofilm activity of AuNCs in vitro

#### Crystal violet assay

*F. nucleatum* suspensions were incubated with AuNCs (0, 0.1, 0.2, and 0.4 mM) on saliva-coated 24-well plates for 48 h to allow the formation of biofilms. The plates were washed three times to remove non-adherent bacteria. Then biofilms were fixed with methanol for 15 min, stained with 0.01% (w/v) crystal violet, and air-dried at room temperature. Finally, the crystals were dissolved sufficiently in 95% ethanol, and absorbance was measured by a microplate reader at 590 nm.

#### EPS degradation within mature biofilms

Hydroxyapatite (HA) discs were coated with filter-sterilized saliva for 1 h at 37 °C to form acquired pellicles. *F. nucleatum* were cultured on the surface of the HA discs for 48 h in medium supplemented with AlexaFluor 647-dextran conjugate (Ex/Em:647/668 nm). After treatment with AuNCs (0.4 mM) or not for 6 h, the biofilms were stained with SYTO 9 to visualize the bacteria. Finally, confocal fluorescence images and 3D volumetric reconstructions were captured to test the influence of the AuNCs on the matrix by quantifying the biomass and thickness of bacteria and EPS, respectively.

#### Bacterial killing within mature biofilms

The *F. nucleatum* biofilms were evaluated by staining with SYTO 9/PI and observed under CFLM in combination with 3D volumetric reconstruction. Similarly, biofilm morphology was observed by SEM. As described above, biofilms were formed on HA discs and treated with AuNCs (0.4 mM) or not for 6 h. Then formed biofilms were fixed with formaldehyde, dehydrated in a graded ethanol series, and then observed by SEM.

#### Penetrability of AuNCs into the biofilms

*F. nucleatum* were incubated in the upper chamber of a Transwell system with 0.4-µm filter membranes for 48 h to allow biofilm formation. Then the biofilms were carefully washed three times to remove any planktonic bacteria. Next AuNCs (0.4 mM) or PBS were added into the upper chamber; simultaneously, *F. nucleatum* were placed in the lower chamber. After treatment for 6 h, the *F. nucleatum* in the lower chamber were diluted and cultured for another 48 h on blood plates to investigate the biofilm penetrability of the AuNCs.

### Antibiofilm effect of AuNCs in vivo

Animal experiments were carried out according to guidelines for the care and use of laboratory animals. A total of 24, six-week-old male C57 BL/6 mice were housed in specific pathogen-free conditions in a 12 h dark/light cycle. Then all mice were randomly divided into four groups (N = 6): control (Con), ligature only (Lig), ligature with *F. nucleatum* inoculation (Fn), and ligature with alternating *F. nucleatum* inoculation and AuNCs applications (AuNCs). In detail, the maxillary bilateral second molars of all mice were ligatured with 5 − 0 silk suture. In the Con mice, once the silk suture was in place, it was quickly removed to eliminate possible trauma during the ligature operation. In the Lig group, the mice were ligatured with silk suture for 2 weeks. In the Fn and AuNCs groups, the sutures were immersed in an *F. nucleatum* bacterial suspension (∼10^9^ CFU/mL) overnight before they were ligatured. Furthermore, 2% sodium carboxymethyl cellulose (NaCMC) polymer (800–1200 mPaּּ·S) was incorporated with *F. nucleatum* suspension, applied topically around the cervical region of maxillary second molars every other day, with neat NaCMC as a control. Next day, mice in AuNCs group were topically treated with 100 µL of AuNCs (0.4 mM) at the same region, with PBS as control. *F. nucleatum* and the AuNCs were applied alternatively for 2 weeks.

After treatment, all mice were euthanasia, and the silk sutures were collected with aseptic scissors, soaked in 400 µL of PBS, and stirred vigorously by ultrasonic oscillation to obtain a bacterial suspension; 20 µL were diluted, spread evenly on agar plates, and cultured for 48 h. The remaining 380 µL was used for 16S rRNA sequencing. The maxillae were aseptically dissected and collected for histological analysis, RT-qPCR assays, immunohistochemical (IHC) staining, and micro-CT scanning. More details are provided in the Supplementary Materials.

### Statistical analysis

Data are expressed as the Mean ± SD. Statistical analysis was performed using GraphPad Prism 8 software using Student’s *t* test or one- or two-way analyses of variance (ANOVAs) as appropriate. Results were considered statistically significant if P < 0.05.

## Results and discussion

### Characterization of AuNCs

**Fig. 1:**
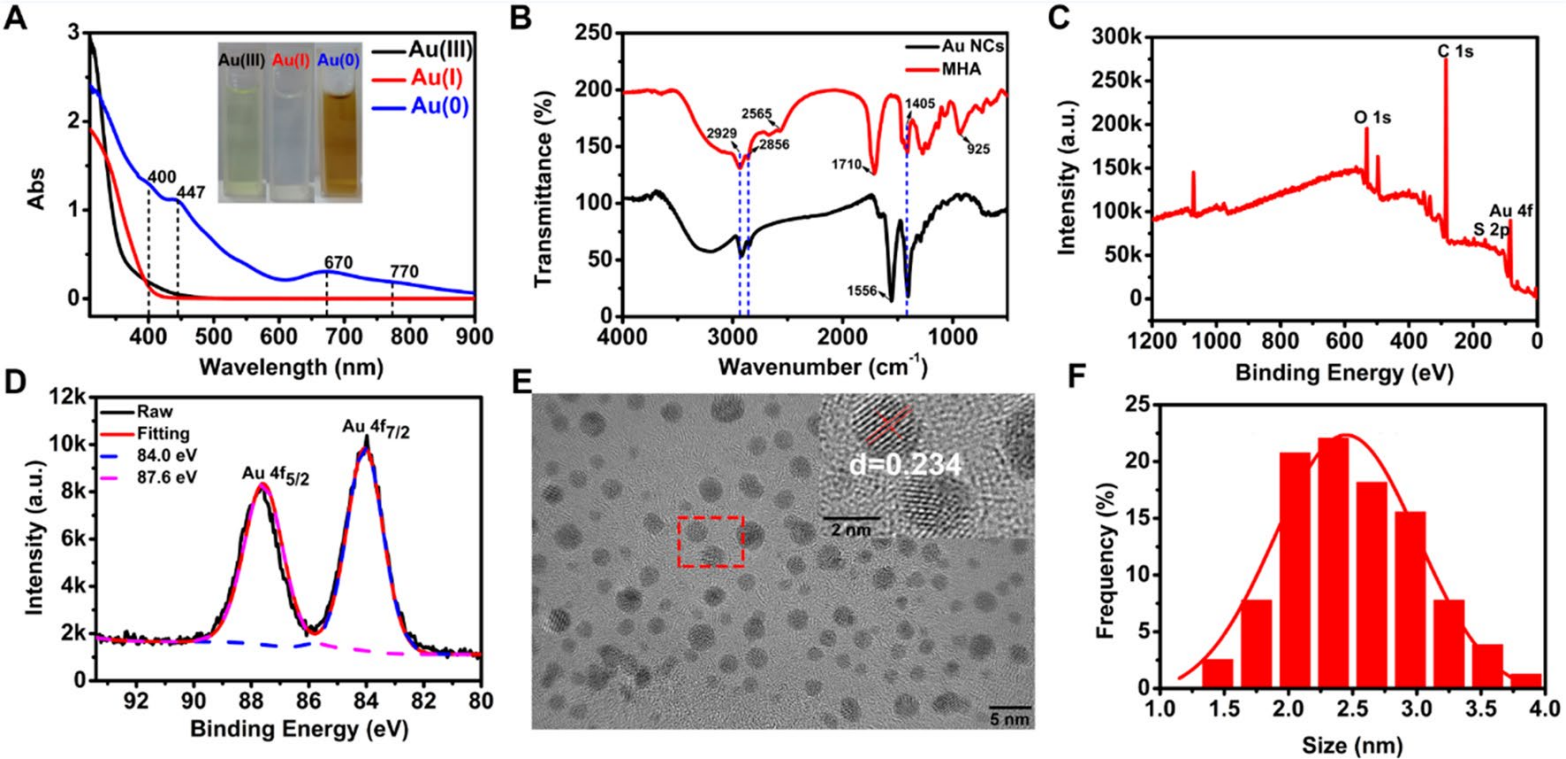
Structure and characterization of the AuNCs. **A** UV-Vis absorption spectrum of solution during preparation; insets indicate the corresponding color changes. **B** FTIR spectrometry of synthesized AuNCs and ligand (MHA). **C** XPS survey spectrum of AuNCs, and **D** the corresponding high-resolution XPS spectrum of Au 4 f. **E** HR-TEM of AuNCs. **F** Size distribution of the AuNCs calculated using HR-TEM.

Different from most previously reported metal nanoparticles, AuNCs of ultra-small size (~ 2 nm) have accurate atomic configurations and geometric structures and are widely used in the biomedical field [31]. In this work, ultra-small nanoclusters with 25 Au atoms (Au_25_NCs) were synthesized according to previous reports [27, 32]. The successful fabrication of Au_25_NCs was characterized by UV-Vis spectrophotometry. As shown in Fig. 1A, the distinct characteristic absorptions of Au_25_NCs occurred at 400, 447, 670, and 770 nm, which is consistent with the reported characteristic absorption peaks of thiolate-protected Au_25_NCs [31]. However, no absorption peaks were observed for Au (III)– and Au (I)–MHA complexes, which were formed by mixing Au (III) and MHA together without a reducing agent. The color of the solution changed from light yellow (Au (III)) to colorless (Au (I)) and finally red–brown (Au (0)), further confirming the formation of Au_25_NCs. Then FTIR spectrometry was used to illustrate the conjugation between Au and thiolate ligands (Au–S–Au). In Fig. 1B, the characteristic band of MHA at 2565 cm^− 1^ is ascribed to the stretching vibration of –SH, which disappeared from the FTIR spectrum of the AuNCs due to the formation of Au–S covalent bonds. Furthermore, the stretching vibration of the carboxyl group (–COOH) at 1710 cm^− 1^ in MHA shifted to 1556 cm^− 1^ in AuNCs, and the out-of-plate deformation of the hydroxyl group (–OH) at 925 cm^− 1^ in MHA disappeared in AuNCs. All these results are attributable to the deprotonation of carboxyl (–COOH) to form carboxylate (–COO^−^) during the reaction. Furthermore, the common peaks at 2929 and 2856 cm^− 1^ are assigned to the stretching and asymmetric stretching vibrations of –CH_2_– in both MHA and AuNCs. The band at 1405 cm^− 1^ is due to the bending vibration of –CH_2_. X-ray photoelectron spectroscopy (XPS) was used to confirm the elemental composition and valence states of AuNCs. As displayed in Fig. 1C, D, the survey scan of AuNCs clearly showed the characteristic peaks of C 1 s, O 1 s, Au 4f, and S 2p, with elemental compositions of 79.92%, 15.30%, 2.06%, and 2.74%, respectively. More importantly, the raw doublet peaks of Au 4f were matched well with the fitting results at 84.0 and 87.6 eV, which are typical of Au 4f_7/2_ and 4f_5/2_, respectively, indicating the successful reduction of Au (III) to Au (0). Finally, HR-TEM was used to characterize the ultra-small structure of AuNCs. As shown in Fig. 1E, the as-synthesized AuNCs exhibited roughly spherical shapes with a homogeneous and well-dispersed distribution, and the fringe of the Au lattice-spacing distance of as-prepared AuNCs was 0.234 nm, which was consistent with the (111) lattice spacing of face-centered cubic (fcc) Au. The histogram of the AuNCs size distribution and the corresponding Gaussian fitting curve is shown in Fig. 1F. The particle sizes of AuNCs range from 1.5 to 4.0 nm with an average diameter of 2.49 ± 0.30 nm. The zeta potential of AuNCs was − 38.8 mV. Overall, the characterizations confirmed that ultra-small Au_25_NCs were successfully prepared by the one-pot reduction synthesis.

### Antibacterial activity of AuNCs against planktonic *F. nucleatum*

**Fig. 2:**
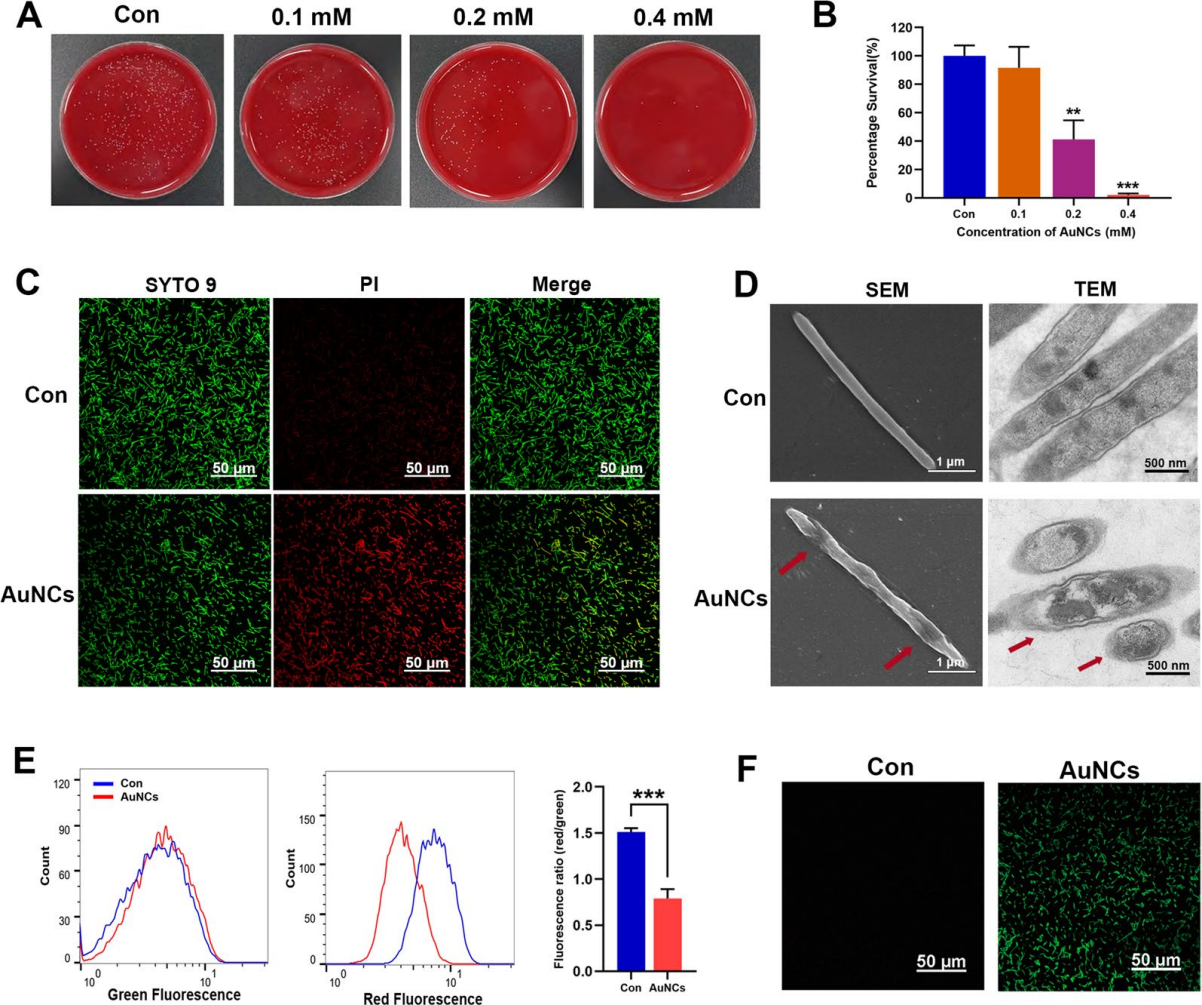
Antibacterial effects and mechanism of the AuNCs. **A**, **B** Photographs and corresponding quantification of *F. nucleatum* on agar plates treated with different concentrations of AuNCs. **C** Images of live/dead fluorescent staining after treatment with AuNCs (0.2 mM), green fluorescence of the SYTO 9 probe indicates all bacteria, and red fluorescence of the PI indicates dead bacteria. **D** SEM and TEM images, **E** membrane potential, and **F** visualization of ROS of *F. nucleatum* treated with AuNCs (AuNCs group, 0.2 mM) or not (Con group). **P < 0.01, ***P < 0.001

The antimicrobial performance of the AuNCs against planktonic *F. nucleatum* was evaluated by a plate-counting assay. As shown in Fig. 2A, B, the viability of *F. nucleatum* demonstrated the dose-dependent antibacterial activity of AuNCs. The growth of bacteria was significantly inhibited by AuNCs at a concentration of 0.2 mM. To evaluate the mechanisms of action by which AuNCs kill bacteria, STYO 9/PI staining was conducted to examine bacterial membrane integrity. All bacteria can be stained by SYTO 9 with green fluorescence, whereas only those cells with damaged membranes can be penetrated by PI to exhibit red fluorescence. As shown in Fig. 2C, the red fluorescence intensity was higher in the AuNC-treated group than in the control group, showing that most bacterial membranes were seriously damaged by AuNCs. Then morphological changes and microstructural damage of bacteria were analyzed by SEM and TEM (Fig. 2D). Normally, *F. nucleatum* are classical spindle-shaped rods with full smooth and intact cell membranes. After incubation with AuNCs, bacterial cell wall integrity was damaged, and the cells had unclear borders. Similar results were also observed by TEM: the cytoplasmic membrane became blurred, the interior structure was lost, and there were large transparent areas in the AuNC-treated group. These results indicated that AuNCs induced the lysis of *F. nucleatum.*

Membrane potential is crucial for bacterial energy metabolism and is an early sign of membrane damage [33]. The fluorescent probe DIOC_2_(3) was used to determine the impact of AuNCs on the membrane potential of *F. nucleatum*. A ratio of the intensities of red and green fluorescence of the probe was calculated. For normal bacteria, the ratio is high due to the large membrane potential, but it will decrease for membrane-damaged bacteria due to dissipations of membrane potentials [34]. As shown in Fig. 2E, the red/green fluorescence ratios were obviously reduced about 50% in the AuNCs group, indicating that AuNCs treatment caused serious damage to cell membranes, which might inhibit bacterial growth via a membrane depolarization mechanism. We further tested ROS generation inside *F. nucleatum*. The results showed that AuNC-treated *F. nucleatum* exhibited strong green fluorescence (Fig. 2F), indicating that ROS levels were significantly enhanced. Bacteria-internalized AuNCs can induce ROS generation and metabolic imbalances [26, 35]. All the above results demonstrated that the as-prepared ultra-small AuNCs had excellent antibacterial effects against *F. nucleatum* by destroying membrane integrity, disrupting membrane potential, and generating ROS. It would be difficult for bacteria to develop resistance to AuNCs based on their antimicrobial mechanisms [36, 37].

### Antibiofilm activities of AuNCs in vitro

**Fig. 3:**
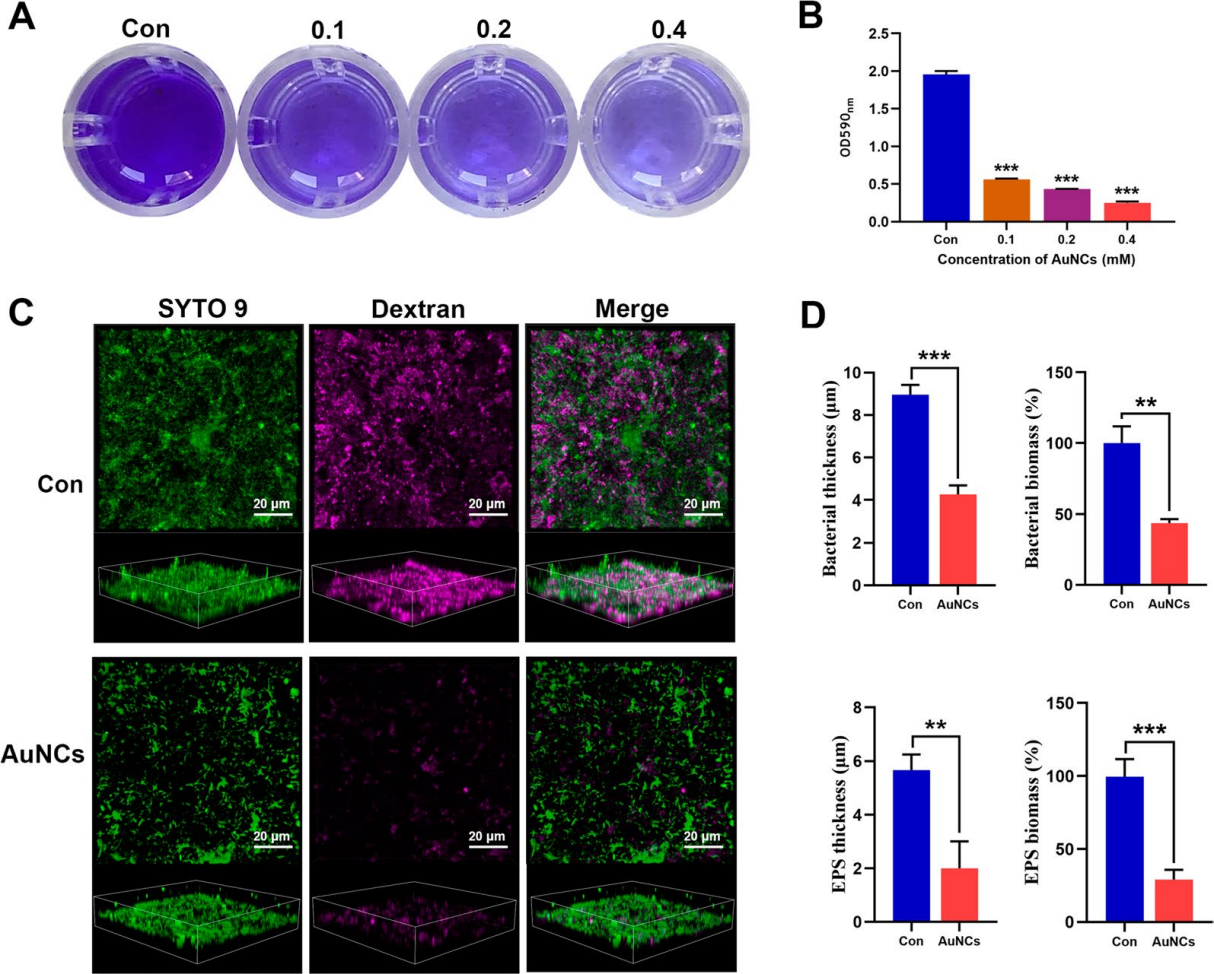
Antibiofilm activity of AuNCs. **A** Inhibitory effects of AuNCs of different concentrations on biofilm formation as determined by crystal violet staining. **B** The semi-quantitation of biofilm biomass by measurement of OD_590_ after crystal violet staining. **C** Confocal 3D image of biofilm treated with AuNCs (0.4 mM). Green fluorescence of the SYTO 9 probe indicates all bacteria, and aubergine fluorescence of the dextran indicates EPS. **D** Quantification of bacteria and EPS. **P < 0.01, ***P < 0.001

Under natural conditions, most oral bacteria exist as biofilms instead of in the planktonic state [2]. Biofilm bacteria are much more recalcitrant to conventional antimicrobials due to their properties different from planktonic bacteria, as well as the low penetration of antimicrobials in biofilms [2, 38]. Initially, we used a semi-quantitative crystal violet assay to observe the inhibition effect of AuNCs on *F. nucleatum* biofilm formation. AuNCs can significantly disrupt biofilm formation in a dose-dependent manner (Fig. 3A, B). When the bacteria were incubated with 0.4 mM AuNCs, the biofilm became sparse, and the biomass rapidly decreased by 87%, indicating that the AuNCs effectively inhibited biofilm formation.

We next investigated whether mature *F. nucleatum* biofilms could be eradicated by AuNCs. The Alexa Fluor 647-labelled dextran conjugate was used to label insoluble EPS in biofilms [39]. As shown in Fig. 3C, the biofilms displayed clusters of densely packed bacterial cells (green fluorescence) embedded by an abundant amount of EPS matrix (aubergine fluorescence) in the control group. After treatment with 0.4 mM AuNCs, the thicknesses of bacteria and EPS were reduced by nearly 50% and 65%, respectively (Fig. 3D). Furthermore, the biomasses of bacteria and EPS were remarkably reduced to around 45% and 30%, respectively (Fig. 3D). In recent years, Au nanoparticles with intrinsic enzyme-mimetic activity have shown great promise in biomedical applications [40]. Ultra-small AuNCs have excellent catalytic activity by providing more exposed active sites [29]. It has been demonstrated that AuNCs possess peroxidase-like activity, and convert hydrogen peroxide into hydroxyl radicals [29, 41]. The hydrogen peroxide would accumulate inside biofilms during the metabolism of *F. nucleatum* [42, 43]. Therefore, AuNCs could induce the generation of ROS in bacteria mainly through their intrinsic oxidase- and peroxidase-like catalysis, which was also confirmed by Zheng et al. [28]. Many previous studies indicated that the generation of hydroxyl radicals could destroy EPS components, including polysaccharides, proteins, and nucleic acids [44, 45]. The above results suggest that AuNCs can efficiently inhibit biofilm formation and eradicate mature biofilms by destroying their structure.

**Fig. 4:**
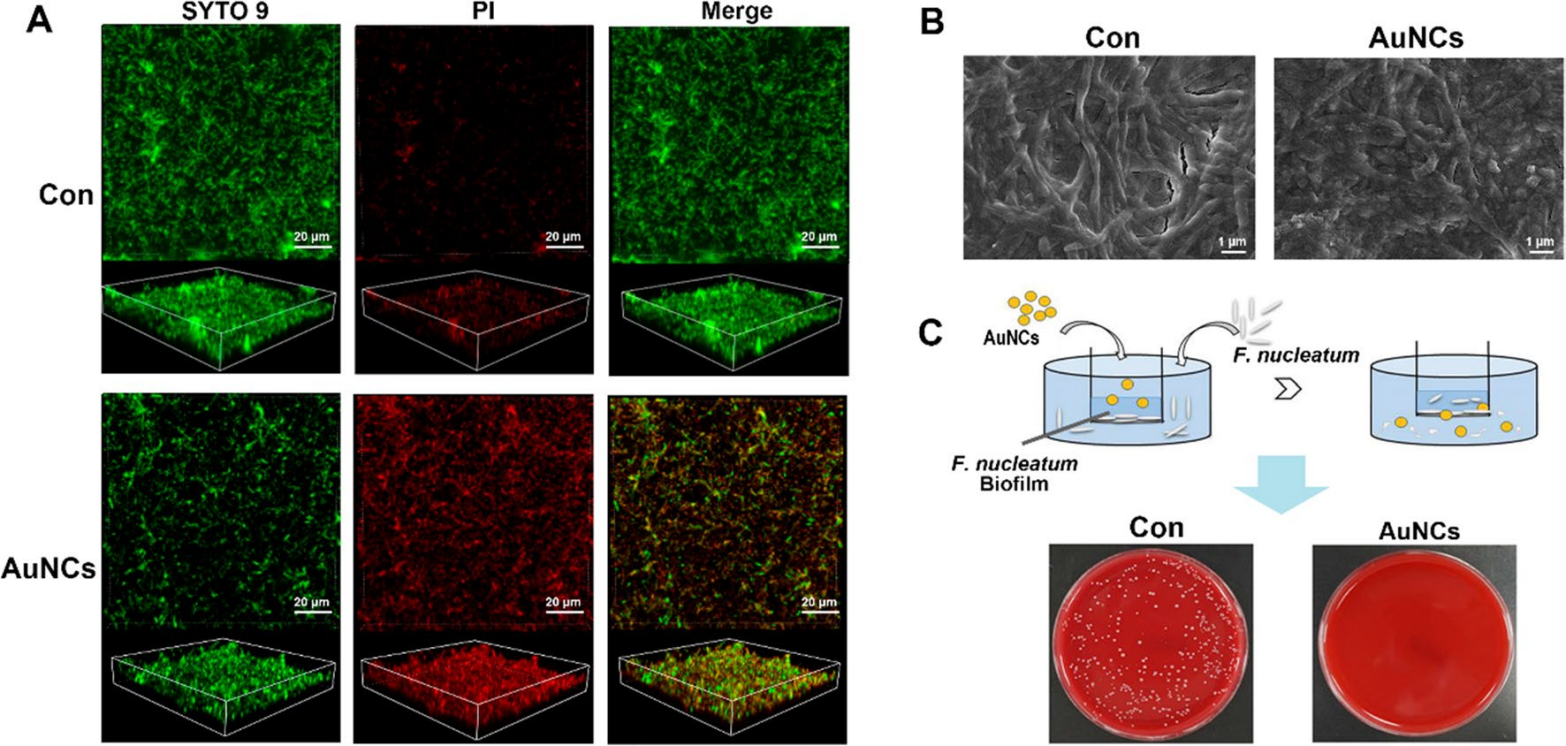
Antibiofilm mechanism of AuNCs. **A** Live/dead fluorescent staining of *F. nucleatum* biofilm treated with AuNCs. **B** Morphological and structural changes of *F. nucleatum* biofilm treated with AuNCs visualized by SEM. **C** Testing the biofilm penetrability of AuNCs and image of bacterial colony forming units from the lower chamber. The concentration of AuNCs was 0.4 mM

Finally, we explored the state of *F. nucleatum* inside biofilms after AuNCs treatment. The biofilms were stained with SYTO 9/PI to evaluate the bacterial viability by 3D reconstruction. Figure 4A shows that the intensity of red fluorescence was enhanced significantly after AuNCs treatment, indicating that the number of bacteria with compromised membranes had increased dramatically. To further investigate the influence of AuNCs on the cellular membrane of *F. nucleatum*, SEM analysis was carried out. As shown in Fig. 4B, most AuNC-treated *F. nucleatum* showed membrane damage, while the controls showed intact and smooth cell membranes. These results suggested that the loss of cell viability was associated with membrane damage, which correlated with the results of PI staining. However, since diffusion is the dominant factor affecting transport in biofilms, the mobility and bioavailability of nanoparticles are heavily influenced by their diffusion coefficients [46]. When trying to disperse established biofilms, slow permeation and diffusion within biofilms largely impact the effects of nanoparticles [47]. Hence, the ability of AuNCs to get through the physical barrier of biofilms was also evaluated. As shown in Fig. 4C, viable bacteria counts in the lower chamber were much lower than for the control group after adding AuNCs into the mature biofilm in the upper chamber. This indicated that AuNCs worked well in penetrating biofilms and exerting bactericidal activity against *F. nucleatum* inside biofilms.

### Antibacterial activity of AuNCs against F. nucleatum in vivo

**Fig. 5:**
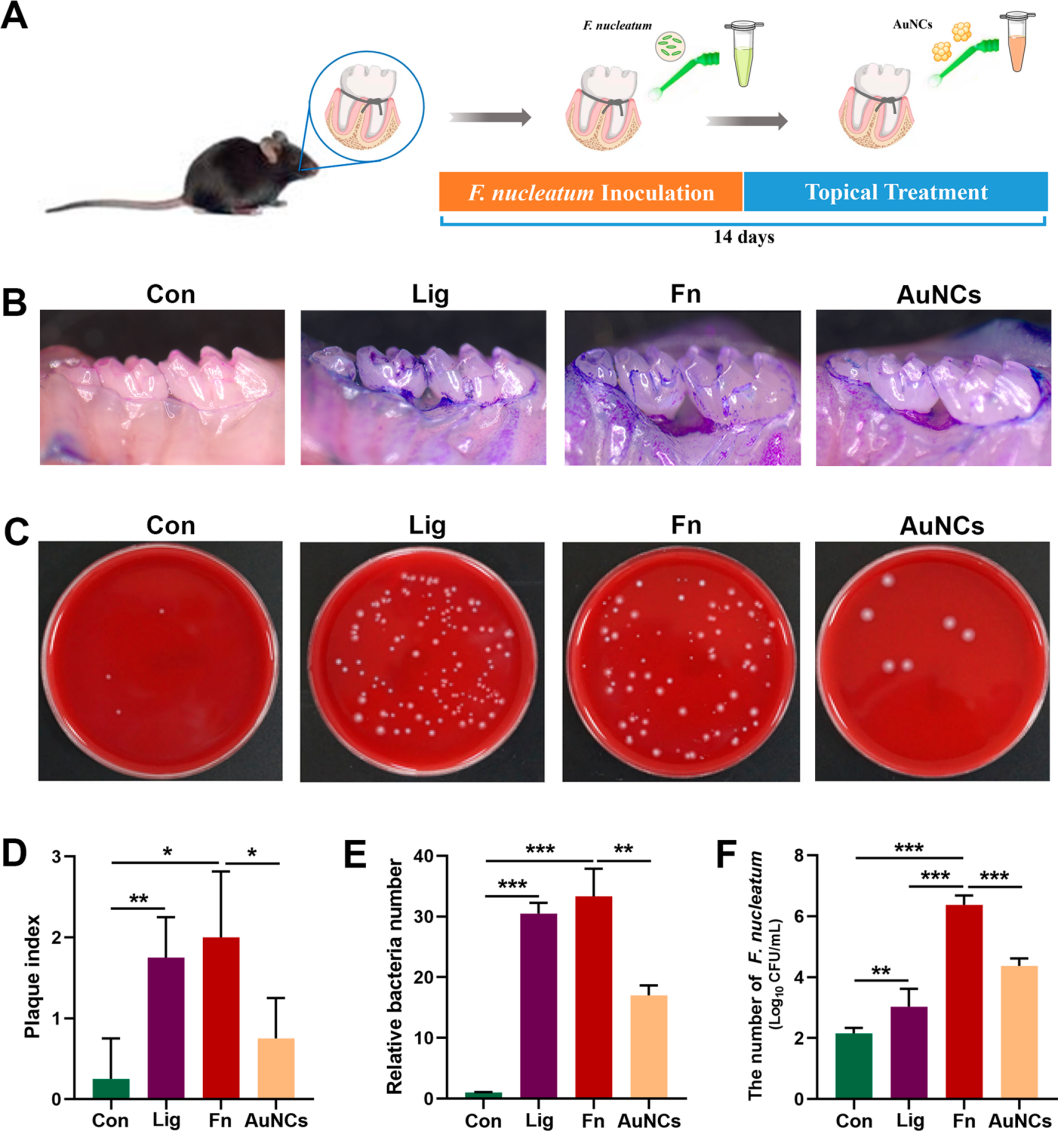
Effects of topical treatment with AuNCs on dental plaque biofilms. **A** Timeline of the *F. nucleatum* infection model and subsequent treatments. **B** Photographs of dental plaque after staining under stereomicroscopy. **C** Photographs of bacterial colony forming units obtained from the biofilms on the silk sutures treated under various experimental conditions. **D** Plaque index. **E** The corresponding quantification of colony forming units. **F** Quantification of *F. nucleatum* according to standard curves, obtained from the biofilms on the silk sutures treated under various experimental conditions. *Con* treatment with PBS, *Lig* ligature and treatment with PBS, *Fn* ligature with *F. nucleatum* inoculation, *AuNCs* ligature with alternate applications of *F. nucleatum* and AuNCs (0.4 mM). *P < 0.05, **P < 0.01, ***P < 0.001

To further investigate the antibiofilm performance of AuNCs in vivo, the accumulation of dental plaque was induced via ligature silk suture placement together with local inoculation of *F. nucleatum* around the molars in a mouse model [48]. Figure 5A outlines the detailed procedure from animal modelling to treatment of dental plaque in vivo. To visually observe therapeutic efficacy, dental plaque staining of the maxillary was performed. The areas of dental plaque in the Lig and Fn groups were larger, which indicated that the ligatures facilitated biofilm formation. After AuNCs treatment, the amount of plaque was significantly reduced (Fig. 5B). The mean plaque index of the AuNCs group was also lower than that of the Fn group (Fig. 5D). The silk sutures around the second molars were collected for further anaerobic bacteria colony counts. As shown in Fig. 5C, the numbers of bacteria were significantly larger in the Lig and Fn groups, while there were fewer bacteria in the AuNCs group. It presumably because AuNCs decreased the amount of *F. nucleatum*. The support for strict anaerobes growth in oxygenated conditions from relatively aerotolerant *F. nucleatum* was diminishing [49]. The quantitative information statistically verified the aforementioned results. As shown in Fig. 5E, the relative bacterial count in the Fn group showed a 33-fold increase compared with the Con group, while the increase was only 17-fold in the AuNCs group. These results indicated that AuNCs could destroy *F. nucleatum*-induced biofilms in vivo. To evaluate the burden of *F. nucleatum* in the periodontal environment after AuNCs treatment, the numbers of *F. nucleatum* in the silk sutures were quantified by RT-qPCR. As shown in Fig. 5F, the number of *F. nucleatum* in the Fn group was three orders of magnitude higher than that of the Lig group, indicating successful colonization of *F. nucleatum* in the oral cavity. After topical treatment with AuNCs, the numbers of *F. nucleatum* were reduced by more than two orders of magnitude (P < 0.001). *F. nucleatum* possess the unique ability to co-aggregate with numerous species of oral microbes, which promote the development of multi-species biofilms [50]. Liu et al. reported that vaccination targeting *F. nucleatum* could abrogated the enhancement of bacterial co-aggregation and biofilm formation [10]. The reduction in *F. nucleatum* may be the underlying cause of smaller amounts of dental plaque in the AuNCs group.

### Alleviation of inflammation and bone loss by AuNCs

**Fig. 6:**
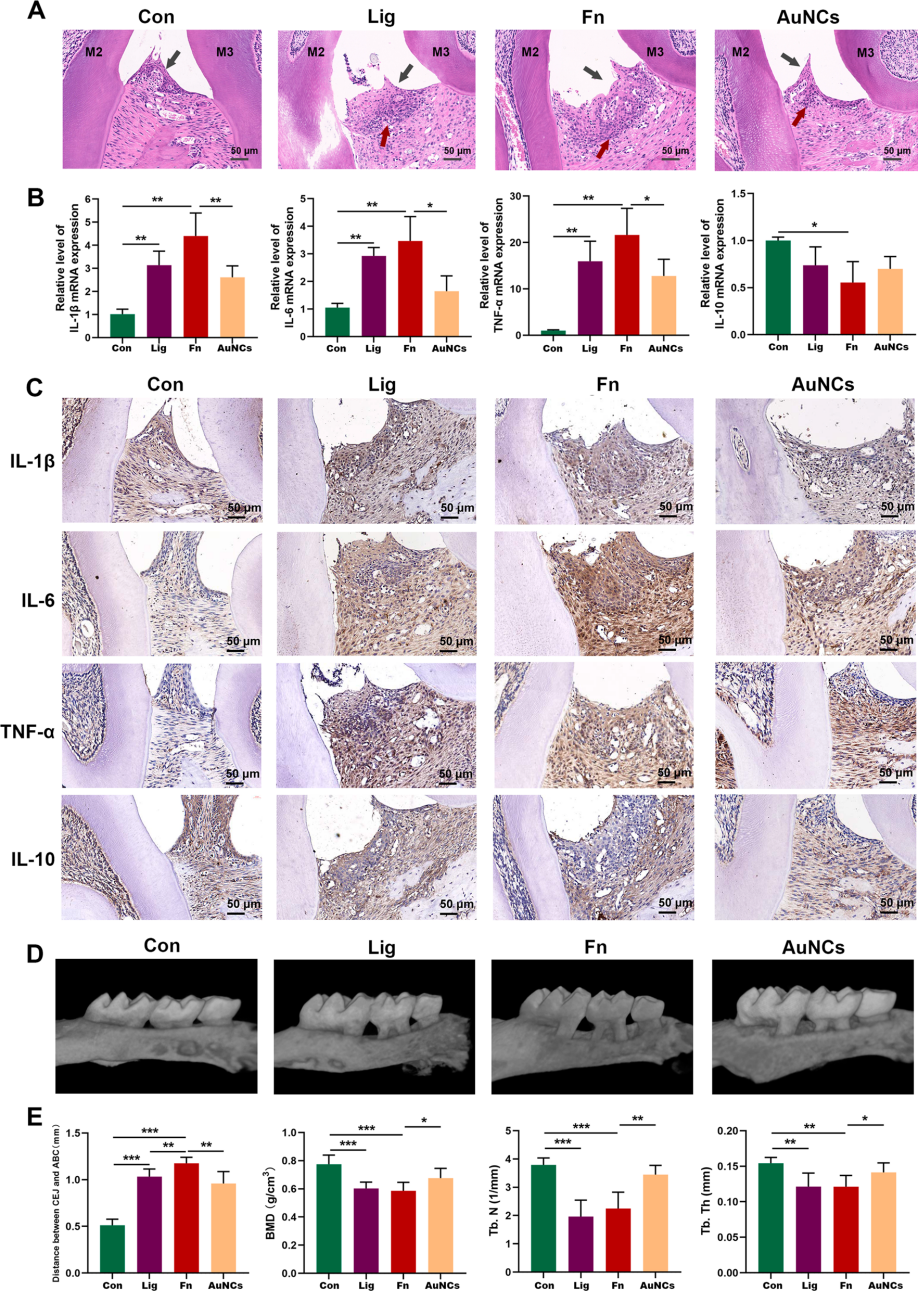
Effects of topical AuNCs treatment on periodontal inflammation and bone loss. **A** H&E staining of tissue sections from the maxillary of mice. Red arrows indicate inflammatory cell infiltration. Black arrows indicate epithelial destruction. M2 indicates the second molar, and M3 indicates the third molar. **B** RT-qPCR analysis of relative mRNA levels, and **C** IHC staining of the protein levels of the inflammatory cytokines IL-1β, IL-6, TNF-α, and IL-10. **D** 3D micro-CT reconstructed images of maxillary molar area. **E** Alveolar bone parameters assessed by micro-CT. *Con* treatment with PBS, *Lig* ligature and treatment with PBS, *Fn* ligature with *F. nucleatum* inoculation, *AuNCs* ligature with alternate applications of *F. nucleatum* and AuNCs (0.4 mM). *P < 0.05, **P < 0.01, ***P < 0.001

Plaque accumulation around a tooth can induce periodontal inflammation and subsequent alveolar bone loss (ABL) [51]. First, we performed haematoxylin and eosin (H&E) staining to evaluate the inflammation response in periodontal tissue. As shown in Fig. 6A, prominent loss of epithelial attachment and infiltration of inflammatory cells were observed in periodontal tissues of the Lig, Fn and AuNCs groups. The presence of histological inflammatory manifestations was most obvious in the Fn group, while it was improved in the AuNCs group with less attachment loss and milder inflammation. Next, the expression levels of inflammatory cytokines were evaluated by RT-qPCR and IHC staining (Fig. 6B, C). Compared with the Fn group, the levels of proinflammatory cytokines interleukin (IL)-1β, tumor necrosis factor (TNF)-α, and IL-6 were significantly reduced after AuNCs treatment, while the expression of IL-10 was slightly elevated. *F. nucleatum* have been demonstrated to promote proinflammatory cytokine expression by immune cell and gingival fibroblasts [6, 7]. In a clinical study, it also has been observed that reduced *F. nucleatum* DNA concentration in dental plaque was associated with the ameliorative periodontal inflammatory symptoms [52]. These results indicated that AuNCs could alleviate the inflammatory response significantly owe to the reduced *F. nucleatum* and its related biofilm in vivo. Then ABL levels were observed by micro-CT. As shown in Fig. 6D, the distance between the cemento–enamel junction (CEJ) and alveolar ridge crest (ARC) represents the degree of ABL. The ABL of the Fn group was the highest among the four groups, and the ABL of the AuNCs group was significantly lower than that of the Fn group. Consistent with this, bone mineral density (BMD), trabecular number (Tb.N), and trabecular thickness (Tb.Th) were significantly lower in the Fn group, while these bone parameters recovered after treatment with AuNCs. These results support the idea that AuNCs ameliorate the course of bone loss in an animal model.

Many studies have employed various antibacterial materials for treatment of oral infectious diseases [39, 53, 54]. Xi et al. designed a multifunctional dual corona vesicle, which was found be effective in disrupting plaque biofilms, thereby significantly alleviating the symptoms of periodontitis [53]. A novel ultra-small Pt nanoclusters (PtNCs) has been found with the similar effect [54]. In these studies, the antibiofilm effects were evaluated on *Escherichia coli* or *Staphylococcus aureus* [53, 54]. By contrast, we focused on the effect of AuNCs against *F. nucleatum*, the key bacteria in oral biofilm, in vitro and in vivo.

**Fig. 7:**
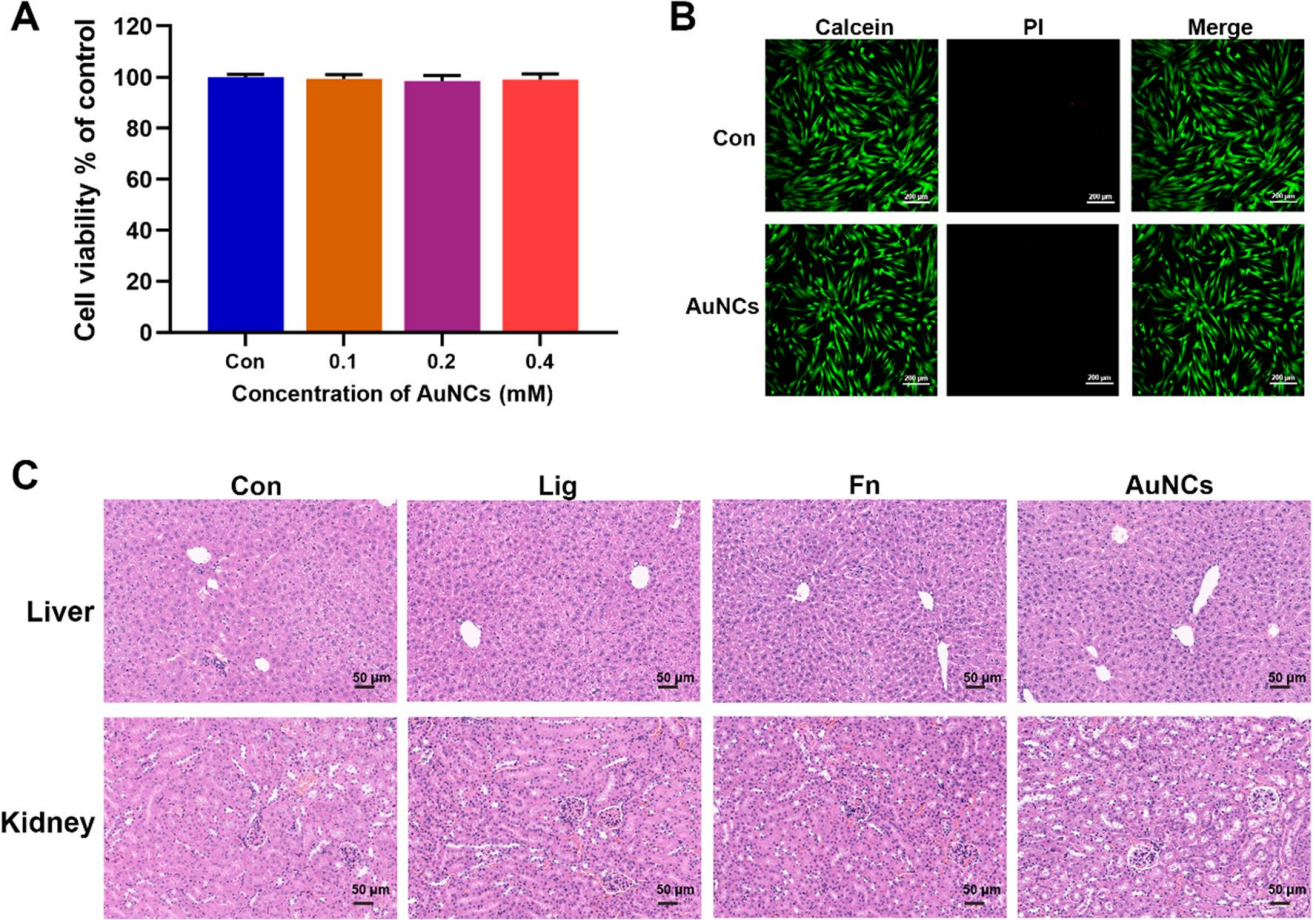
Biocompatibility of AuNCs. **A** The viability of PDLCs treated with AuNCs of different concentrations was measured by CCK-8 at 48 h. **B** Live/dead staining of PDLCs treated with AuNCs (0.4 mM) for 48 h. The green fluorescence of the calcein probe indicates live cells, and red fluorescence of PI indicates dead cells. **C** H&E-stained images of liver and kidney collected from mice after different treatments. *Con* treatment with PBS, *Lig* ligature and treatment with PBS, *Fn* ligature with *F. nucleatum* inoculation, *AuNCs* ligature with alternate applications of *F. nucleatum* and AuNCs (0.4 mM)

The biocompatibility of AuNCs was further evaluated. Periodontal ligament cells (PDLCs) are elongated cell types in periodontal tissue and have important matrix remodeling and signaling functions [55]. The cytotoxicity of AuNCs on PDLCs was observed by CCK-8 assays and live/dead cell staining. There was no obvious cell activity decrease or death after AuNCs treatment at effective antibiofilm concentrations (0.4 mM) (Fig. 7A, B), demonstrating that AuNCs were non-toxic for local tissues in the oral cavity. Furthermore, H&E staining of the liver and kidney showed no assessable damage or abnormalities after AuNCs treatment (Fig. 7C), which indicated that AuNCs have no obvious systemic toxicity. Many studies have also reported that AuNCs of average size < 2 nm have shown good biocompatibility in vivo [56]. AuNCs are rapidly excreted through the kidneys, while nanoparticles > 6–8 nm were easily retained [25, 57]. This rapid renal clearance could greatly reduce the potential for long-term toxicity [58].

### Effects of AuNCs on oral and gut microbiota

**Fig. 8:**
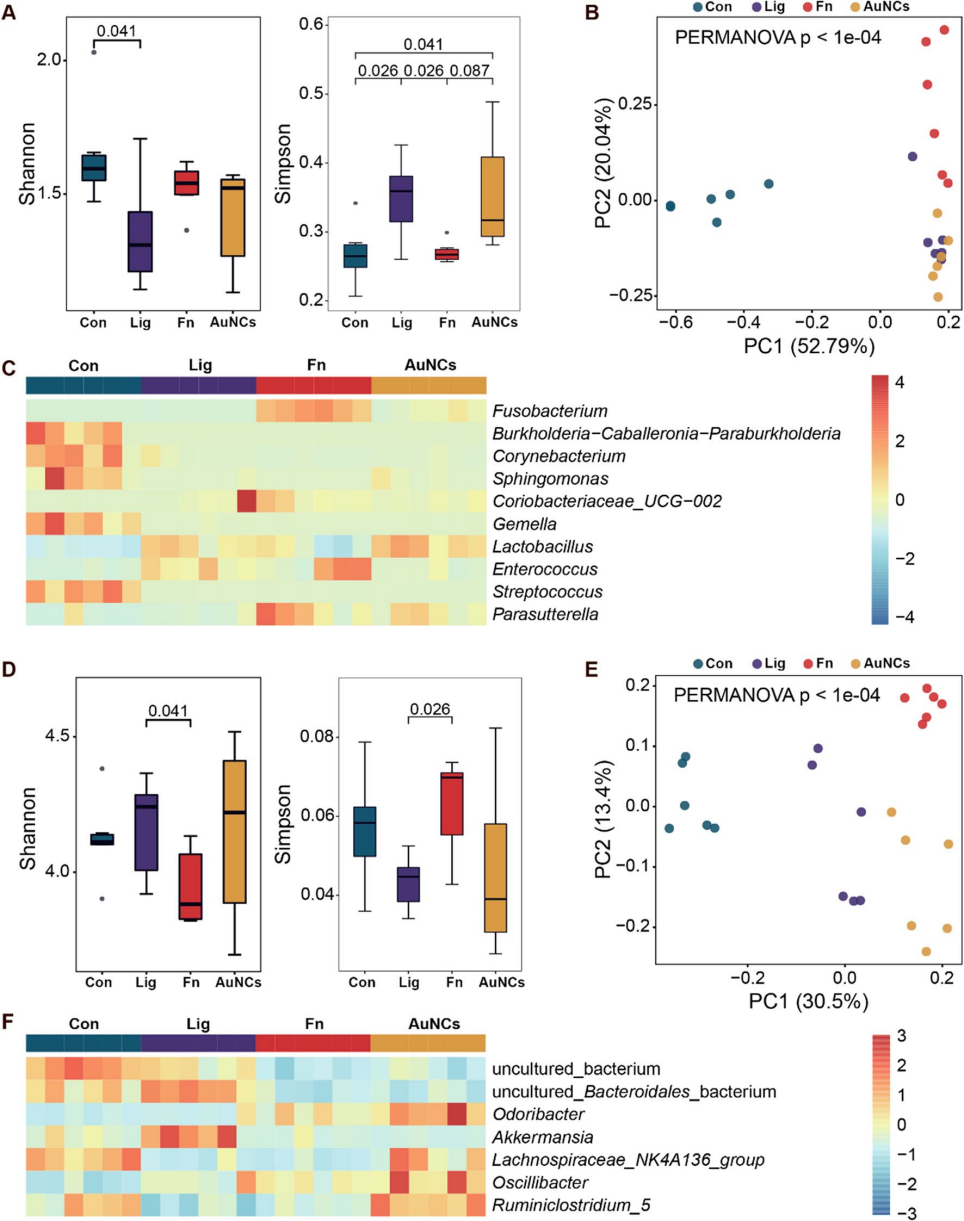
Effects of oral treatment with AuNCs on oral and gut microbiomes. (**A**/**D**) Richness and diversity of microbiome among groups in oral and gut, respectively. (**B**/**E**) Principal coordinate analysis (PCoA) using the Bray–Curtis distance was conducted to reveal the microbiome compositions among groups. (**C**/**F**) Relative abundances of differential bacterial genera across all samples were centered and scaled in the row direction and plotted using a heatmap. *Con* treatment with PBS, *Lig* ligature and treatment with PBS, *Fn* ligature with *F. nucleatum* inoculation, *AuNCs* ligature with alternate applications of *F. nucleatum* and AuNCs (0.4 mM)

**Fig. 9:**
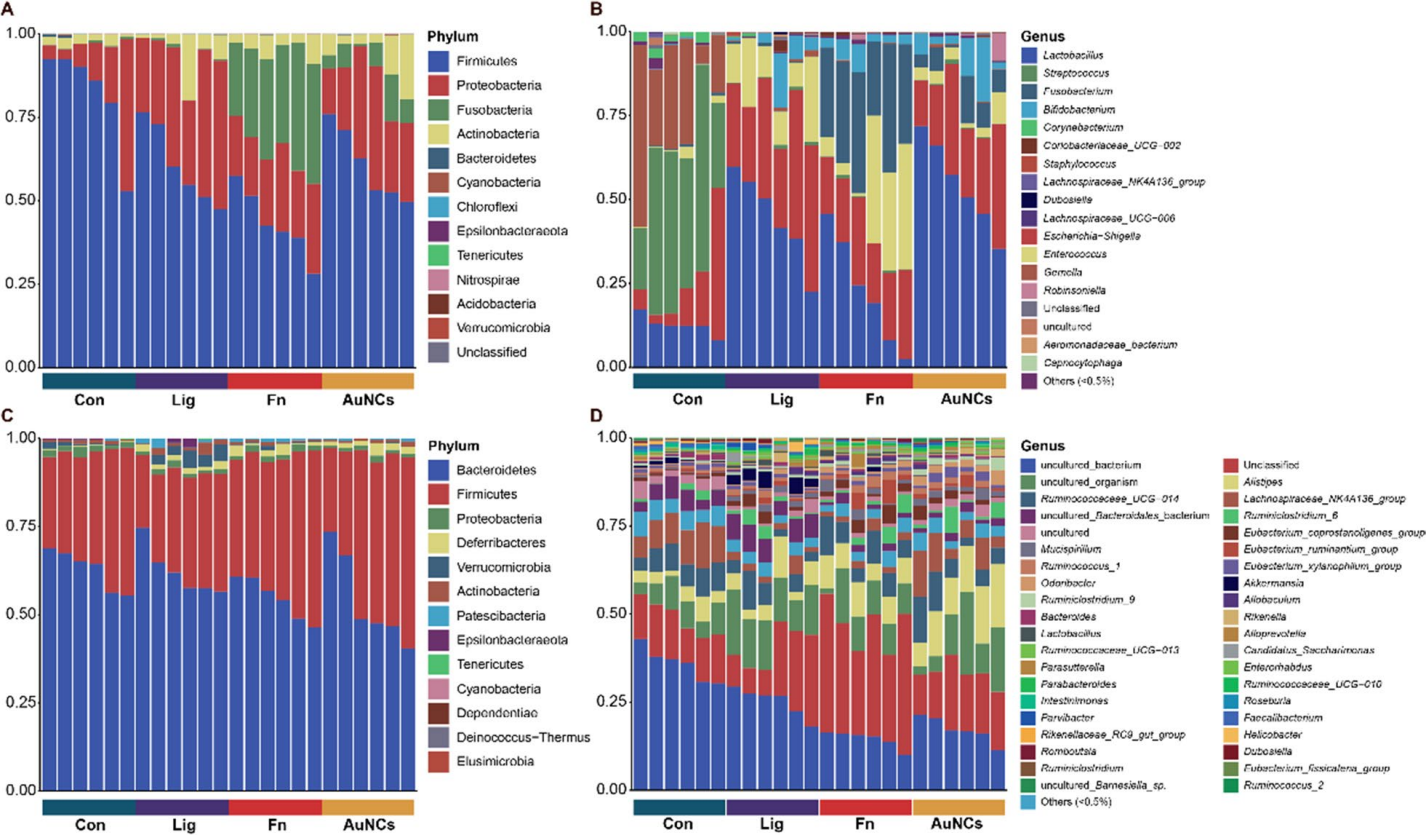
Oral and gut microbiome taxonomic composition histograms of each sample are shown at phylum (**A**/**C**) and genus (**B**/**D**) levels separately. At the phylum level, all bacteria were used to draw the bar graph. At the genus level, bacteria with relative abundances less than 0.5% in all samples were classified as ‘others’. *Con* treatment with PBS, *Lig* ligature and treatment with PBS, *Fn* ligature with *F. nucleatum* inoculation, *AuNCs* ligature with alternate applications of *F. nucleatum* and AuNCs (0.4 mM)

**Fig. 10:**
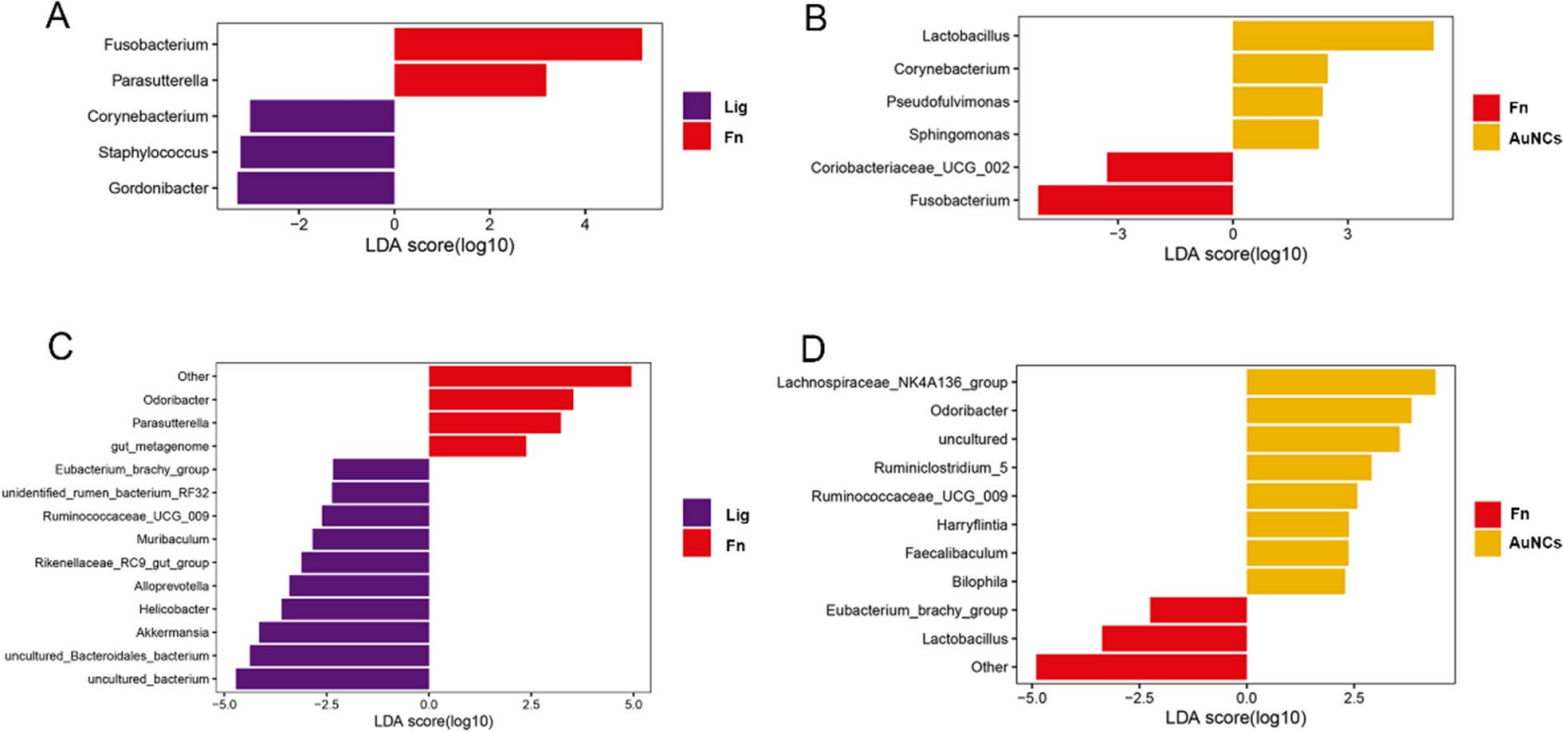
LefSe plots of differentially abundant genera in the oral (upper) and gut (lower). The microbiomes of comparisons between groups Lig vs. Fn (**A**/**C**) or AuNCs vs. Fn (**B**/**D**). Higher linear discriminant analysis (LDA) scores indicate greater effect sizes (log_10_LDA score > 2.0)

To evaluate the overall effects of AuNCs on oral microbiota, sequencing of the microbiome of the silk sutures was performed. The α-diversity of the microbiota was estimated using Shannon and Simpson indexes; a higher Shannon index and lower Simpson index indicated higher α-diversity. The results showed that the Shannon index increased and the Simpson index decreased in the Fn group compared with the Lig group (Fig. 8A). The higher α-diversity in the Fn group may be due to greater pathogenic bacterial adhesion induced by *F. nucleatum* [5]. After AuNCs treatment, the α-diversity of the microbiota declined. Similarly, principal coordinate analysis (PCoA) showed that the oral bacterial composition of the Lig and Fn groups formed two distinct clusters, whereas the AuNCs group had a composition more similar to that of the Lig group than that of the Fn group (Fig. 8B). These results suggested that AuNCs could partly improve the oral microbiota disorder caused by *F. nucleatum*. A random forest heat map also showed that the abundance of *Fusobacterium* increased in the Fn group but obviously decreased in the AuNCs group (Fig. 8C). This phenomenon indicated that AuNCs could eliminate or kill *F. nucleatum* in dental plaque biofilms in accordance with the quantification of *F. nucleatum* by RT-qPCR. The detailed analysis of the structure of the oral microbiota at the phylum and genus levels is illustrated in Fig. 9A, B. In all groups, the most abundant phyla were Firmicutes and Proteobacteria. The abundance of *Fusobacterium* was increased in the Fn group and decreased in the AuNCs group. The linear discriminant analysis effect size (LefSe) analysis was applied to identify specific genera that differed in abundance between two groups. Compared with the Lig group, the abundances of *Fusobacterium* and *Parasutterlla* were higher in the Fn group, whereas *Corynebacterium*, *Staphylococcus*, and *Gordonibacter* abundances were lower (Fig. 10A). After AuNCs treatment, *Lactobacillus*, *Corynebacterium*, *Pseudofulvimonas*, and *Sphingomonas* increased, whereas *Coriobacteriaceae_UCG_002* and *Fusobacterium* decreased (Fig. 10B). Specifically, the abundance of probiotic *Lactobacillus* significantly increased in the AuNCs group.

Biofilms are microbial communities and complex interactions exist among different microorganisms [59, 60]. The expansion or enhanced virulence of pathogenic populations can lead to decrease of some microbiotas, such as probiotics [60]. AuNCs may reduce this inhibitory effect of pathogenic bacteria by killing *F. nucleatum*. *Lactobacillus* species were widely studies for oral diseases treatments in clinical studies and animal experiments [61]. It have been identified that *Lactobacillus* can reduce the count of oral pathogenic bacteria and inhibit biofilm formation [5, 62]. In another study, Naha et al., also evaluated the influence of dextran-coated iron oxide nanoparticles (Dex-NZM) on oral microbiota, when they were used to control tooth decay related biofilm [39]. They found that there no significant changes of oral microbial composition and diversity after Dex-NZM treatment [39]. The inconsistency may due to the differences in ecological niches between tooth enamel and periodontal tissue.

We further analyzed the changes in the gut microbiota community structure. The α-diversity decreased significantly in the Fn group compared with the Lig group (Fig. 8D). PCoA showed that the gut bacterial composition of the Lig and Fn groups were clearly clustered and separated (Fig. 8E). These results demonstrated that *F. nucleatum* colonization in the oral cavity affected the gut microbiota. Increasing evidence has shown that dysbiotic oral microbiota can mediate systemic diseases by disturbing the gut microbiota [63]. However, AuNCs reversed the influence of oral *F. nucleatum* inoculation on the gut microbiota as shown by α-diversity and PCoA analysis (Fig. 8D, E). The dominant phyla and genera are shown in Fig. 9. At the phylum level, Bacteroidetes and Firmicutes constituted more than 90% of the gut bacterial community in all groups. The Firmicutes/Bacteroidetes ratio plays a crucial role in maintaining the microbial balance of the gut [64]. This ratio was reduced in the Fn group compared with the Lig group, while AuNCs treatment restored the ratio. These results demonstrated that reduction of *F. nucleatum* by AuNCs treatment in the oral cavity may contribute to the restoration of gut microbiota balance. The changes in specific bacteria between two groups were also identified by LefSe analysis (Fig. 10C, D). Some previous studies have been concerned about the influence of nanoparticles applied in oral cavity on gut microbiota. Li et al. applied modified AuNPs for therapy of bacterial infection induced by *E. coli* in the gut and found that oral administration of AuNPs can cure infection in mice without compromising intestinal microflora [65]. Meanwhile, AuNPs could increase the relative abundance of typical probiotics (*Akkermansia* and *Bifidobacterium*) after long-term administration [65]. Chen et al. compared the alterations of gut microbiota composition after oral exposure with different metal nanoparticles [66]. It was found that AgNPs ingestion reduced the Firmicutes/Bacteroidetes ratio, increased the lowly abundant families of bacteria, and decreased the probiotic bacteria genus *Lactobacillus*. SiO_2_NPs increased gut microbial species richness and diversity and an obvious increase of the genus *Lactobacillus* was recorded. No obvious disturbance of gut microbiota was found in mice that ingested TiO_2_NPs [66]. These results demonstrated that nanoparticles applied in oral cavity could have an influence on gut microbiota, which is associated with the characteristics of nanoparticles. Overall, AuNCs treatment in the oral cavity did not induce a dramatic change of gut microbiota at specific species in our study, considering the high complexity of the gut microecosystem.

## Conclusion and perspectives

Altogether, AuNCs have promise for the prevention and treatment of biofilm-related oral diseases. They may be incorporated into the toothbrush bristles to enhance the effect of mechanical plaque removal. Oral hygiene products, such as toothpastes and mouthwash solutions were also added with AuNCs to reduce the formation of oral biofilm. In addition, AuNCs gel may improve the therapeutic effect of periodontitis and peri-implantitis. However, many translational hurdles need to be solved before clinical application. First, a rigorous assessment of AuNCs’ toxicity and impact on other organs is needed. The main clearance route of oral topical application is via ingestion, which may result in systemic influence. Second, there are many other typical oral pathogenic bacteria, such as *P. gingivalis* and *A. actinomycetemcomitans*. The antimicrobial effects of AuNCs on these bacteria should be studied. In addition, specificity also is an important factor in clinical applications. The antibodies or peptides could be considered to distinguish between pathogenic and commensal bacteria, which enhance the specificity of AuNCs and reduce the impact on commensals. Future studies should focus on enhancing the therapeutic effect and specificity, while minimizing toxicity.

In conclusion, we presented ultra-small AuNCs as an effective antibiofilm agent for dental plaque. AuNCs exhibited antibacterial activity against *F. nucleatum* through multiple antimicrobial mechanisms, including membrane structural damage, membrane potential change, and ROS generation. Due to their excellent penetration of biofilms, AuNCs killed bacteria inside mature biofilms and promoted degradation of the EPS matrix. The in vivo results showed that topical treatment with AuNCs could disrupt the *F. nucleatum-*induced biofilms, alleviating periodontal inflammation and bone loss. Furthermore, microbiome analyses indicated that AuNCs could restore the disruptions of the oral and gut microbiota caused by *F. nucleatum* colonization. Overall, these results revealed that AuNCs are a promising antibiofilm agent and have a potential for use in the treatment of dental plaque.

## Supplementary Information

**Additional file 1: Table S1.** Primer sequences. Additional sections.

## Data Availability

The data used to support the findings of this study are available from the corresponding author upon request.
