# Supplementary material for: Antibiofilm activity of ultra-small gold nanoclusters against Fusobacterium nucleatum in dental plaque biofilms
Source: J Nanobiotechnology. 2022 Nov 3;20:470. doi: 10.1186/s12951-022-01672-7 (PMC9632159; doi:10.1186/s12951-022-01672-7)
Supplement: Supplementary file 1 — Additional file 1: Table S1. Primer sequences. Additional sections. [file 12951_2022_1672_MOESM1_ESM.docx]

**Additional file 1**

**Antibiofilm activity of ultra-small gold nanoclusters against *Fusobacterium nucleatum* in dental plaque biofilms**

**Materials**

*F. nucleatum* were obtained from the Guangdong Microbial Culture Collection Center (GDMCC; Guangzhou, China). Human periodontal ligament cells (PDLCs) were purchased from ScienCell Research Laboratories (Carlsbad, CA, USA). All chemicals used for the synthesis of AuNCs were obtained from Sigma–Aldrich (St. Louis, MO, USA). The LIVE/DEAD BacLight Bacterial Viability Kits, the fluorescent probe DCFH-DA, and the Alexa Fluor 647–dextran conjugate were obtained from Invitrogen (Carlsbad, CA, USA). The DiOC_2_(3) dye was purchased from MaoKang Biotechnology (Shanghai, China). The crystal violet stain was purchased from Beyotime Biotechnology (Shanghai, China). The Plaque-Check Gel BR was obtained from GC, Inc. (Tokyo, Japan). The total RNA of the gingiva was extracted using TRIzol obtained from Invitrogen (Carlsbad, CA, USA), and cDNA was prepared using the PrimeScript RT Reagent Kit from Takara Biotechnology (Otsu, Japan). IL-1β and IL-10 antibodies were obtained from Proteintech (Wuhan, China). TNF-β and IL-6 antibodies were obtained from ABclonal (Wuhan, China). The genomic DNA of bacteria was extracted using the DNeasy Blood & Tissue Kit from Qiagen (Germantown, MD, USA). Cell Counting Kit-8 and Calcein/PI dye were purchased from Dojindo Molecular Technologies, Inc. (Tokyo, Japan).

**Bacterial culture**

Freshly recovered *F. nucleatum* was cultured in brain heart infusion (BHI) broth medium supplemented with yeast extract (5 g/L), hemin (5 μg/mL), and vitamin K1 (1 μg/mL) in an anaerobic chamber with an anaerobic-gas-producing bag at 37°C. For plate counting, the bacterial suspensions were diluted with sterile PBS (pH 7.4, 10 mM) and then streaked on Columbia blood agar plates supplemented with hemin (5 μg/mL), vitamin K1 (1 μg/mL), and 5% sterile defibrinated sheep blood to allow bacterial growth at 37°C under the same culture conditions.

**Dental plaque staining**

The maxillaries were stained with plaque-check gel for 2 min, carefully washed with PBS to remove superfluous staining, and photos were taken under a stereoscopic microscope to visualize the dental plaques. The plaque index (PLI) was used to evaluate the area and depth of the purple–red staining on the surface of the second molar (score 0–3).

**Quantification of F. nucleatum**

After bacterial DNA was isolated as above, the bacterial load of *F. nucleatum* on the ligature was further quantified by quantitative real-time PCR (RT-qPCR). The primer sequences for the *F. nucleatum* 16S rRNA gene are listed in Table S1, and the obtained Ct values were converted into gene copy numbers and quantified according to the standard curve for *F. nucleatum*.

**Histological analysis**

The maxillaries were stained with haematoxylin and eosin (H&E) for histological evaluation after being fixed, decalcified, dehydrated, transparentised, embedded, and sectioned to a thickness of 4 µm in the mesial–distal direction. Then, the sections were mounted and scanned by a light microscope with a panoramic MIDI viewer (3D HISTECH Ltd. EU, Budapest, Hungary). The livers and kidneys were also stained with H&E according to the above method, except decalcification was unnecessary.

**RT-qPCR assay**

The total mRNA of gingiva was extracted and reverse-transcribed into cDNA according to the manufacturer’s instructions. Then the inflammatory cytokines interleukin (IL)-1β, IL-6, tumor necrosis factor (TNF)-α, and IL-10 were amplified by RT-qPCR using the primer sequences listed in Table S1 and normalized to the housekeeping gene β-actin.

**Immunohistochemical (IHC) staining**

IHC was performed to evaluate the inflammation levels of gingival tissue by the following procedures: dewaxing, inactivation, antigen retrieval, 5% bovine-serum-albumin antigen blocking, and incubation with diluted antibodies IL-1β, IL-6, TNF-α, and IL-10 at 4℃ overnight. Finally, slices were incubated with horseradish peroxidase-labelled goat anti-rabbit/mouse secondary antibody for 30 min at room temperature using DAB as the chromogen.

**Micro-computed tomography**

The maxillaries were scanned by micro-computed tomography (SkyScan 1176; Bruker, Kontich, Belgium) at a voxel resolution of 18 μm. The maxillaries were also three-dimensionally reconstructed using CTvox software and Data Viewer software. Bone loss around the second molars was obtained by measuring the distance from the cement–enamel junction (CEJ) to the alveolar bone crest (ABC) at four sites (mesial and distal sites and central sites in the buccal/palatal sides). The sum of the four distances obtained for each mouse was regarded as alveolar bone loss (ABL). Volumetric measurement was performed after the selection of a three-dimensional region of interest (ROI) around the second molar. The bone mineral density (BMD), trabecular thickness (Tb.Th), and trabecular number (Tb.N) of the ROI were calculated.

**Cytocompatibility assay**

PDLCs were seeded in a 96-well plate and cultured with AuNCs at different concentrations (0, 0.1, 0.2, and 0.4 mM). After 48 h, CCK-8 reagent was added into every well and incubated for 4 h. The absorbance was measured by a microplate spectrophotometer at 450 nm. Cell viability was expressed as a percentage relative to the control groups. For live/dead staining, AuNCs (0.4 mM) were added to the PDLCs for 48 h. Then calcein and PI were added into the above samples, and they were incubated in the dark for another 20 min. Then the cells were observed by CFLM.

**16S rRNA sequencing**

DNA extraction and 16S ribosomal RNA amplicon sequencing

Total genomic DNA from caecal content was extracted by Novogene Bioinformatics Technology (Beijing, China) using the SDS method. DNA concentration and purity was monitored on 1% agarose gels. DNA was diluted to 1 ng/µL using sterile water. The V3–V4 regions of the 16S ribosomal RNA genes were amplified using the specific primer pair 341F/806R with barcoding. Sequencing libraries were generated using the TruSeq® DNA PCR-Free Sample Preparation Kit (Illumina, San Diego, CA, USA) following the manufacturer's recommendations, and index codes were added. The library quality was assessed on the Qubit@ 2.0 Fluorometer (Thermo Fisher Scientific) and the Agilent Bioanalyzer 2100 system (Agilent Technologies Inc., Santa Clara, CA, USA). At last, the library was sequenced on an Illumina NovaSeq platform, and 250-bp paired-end reads were generated.

Processing of 16S rRNA sequencing data

Raw sequencing data obtained from Novogene were assigned to samples based on their unique barcode and truncated by cutting off the barcode and primer sequence. Paired-end reads were merged using FLASH (v1.2.11) with default parameters. The resulting concatenated reads were quality controlled through Trimmomatic (v0.39) in single-end mode with the following settings: “LEADING:3 TRAILING:3 SLIDINGINDOW:4:20 MINLEN:200”. Then, clean reads were imported into, and analysed by QIIME 2 (2020.2) using the deblur algorithm with the minimal read length set to 400 bp. Taxonomic annotation of deblur denoised reads was performed with the q2-classifier plugin using the “classify-sklearn” command based on the SILVA database (v132). Phylogenetic trees were constructed using q2-phylogeny by the “align-to-tree-mafft-fasttree” methods. The Wilcoxon rank-sum test was used to discriminate significant differences in alpha-diversity indices between groups, and P<0.05 was significant. Principal coordinate analysis (PCoA) using the Bray–Curtis distance was used to reveal microbiome composition among groups and was tested with 9999 permutations. The Wilcoxon rank-sum test or Kruskal–Wallis test was performed to find differential bacterial genera, when appropriate, with the Benjamini–Hochberg false discovery rate correction. Adjusted P<0.05 was considered statistically significant.

**Additional file tables**

Table S1 Primer sequences

| **Gene** | **Forward Primer sequences (5’-3’)** | **Reverse Primer sequences (5’-3’)** |
| --- | --- | --- |
| *F. nucleatum*  *16S rRNA* | AAGCGCGTCTAGGTGGTTATGT | TGTAGTTCCGCTTACCTCTCCAG |
| *IL-1β* | AAGGAGAACCAAGCAACGACAAAA | TGGGGAACTCTGCAGACTCAAACT |
| *IL-6* | AGTTGCCTTCTTGGGACTGA | TCCACGATTTCCCAGAGAAC |
| *TNF-α* | TCTTCTCATTCCTGCTTGTGG | GAGGCCATTTGGGAACTTCT |
| *IL-10* | GCCAGAGCCACATGCTCCTA | GATAAGGCTTGGCAACCCAAGTAA |
| *β-actin* | GGTGTGATGGTGGGAATGGG | ACGGTTGGCCTTAGGGTTCAG |
